# Is intralymphatic immunotherapy effective and safe for allergic rhinitis?: A meta-analysis

**DOI:** 10.1097/MD.0000000000040589

**Published:** 2024-11-15

**Authors:** Liangrong Liu, Yacheng Liang, Le Yan, Zhiyong Li

**Affiliations:** a Department of Otorhinolaryngology Head and Neck Surgery, Nanchong Central Hospital, The Second Clinical Medical College, Nanchong, China; b School of Medical and Life Sciences/Reproductive & Women-Children Hospital, Chengdu University of Traditional Chinese Medicine, Chengdu, China.

**Keywords:** allergic rhinitis, immunotherapy, intralymphatic, meta-analysis

## Abstract

**Background:**

As there is much controversy in using intralymphatic immunotherapy (ILIT) as a therapeutic means for allergic rhinitis (AR), its efficacy and safety for AR were investigated based on a systematic review and meta-analysis.

**Methods:**

Databases PubMed, Embase, Cochrane library, and Web of Science were employed to retrieve relevant randomized control studies on ILIT for AR. The search deadline was September 15, 2023. Meta-analysis was performed on the data of the included literature using Stata 15.0.

**Results:**

Eleven randomized control studies were included involving a total of 406 patients. Meta-analysis results revealed that ILIT improved patients’ quality of life [standardized mean difference (SMD) = ‐0.53, 95% confidence interval (CI) = (‐1.00, ‐0.050)], and reduced the adverse events of nasal symptoms [risk ratio (RR) = 0.16, 95% CI = (0.06, 0.45)] as compared to control, whereas no significant difference was discovered in symptom score [SMD = 0.14, 95% CI = (‐0.34, 0.62)], IgE [SMD = 0.93, 95% CI = (‐0.44, 2.30)], medication scores [SMD = 1.37, 95% CI = (‐0.45, 3.18)], comprehensive symptom and medication scores [SMD = 0.93, 95% CI = (‐0.62, 2.47)], nasal symptoms [RR = 0.16, 95% CI = (0.06, 0.45)], and lymphadenectasis [RR = 2.27, 95% CI = (0.37, 6.73)] versus control.

**Conclusion:**

After the application of the ILIT strategy against AR, the quality of life of patients was improved and the incidence of adverse events associated with nasal symptoms was reduced, but the conclusion needed further verification with more high-quality research.

## 1. Introduction

Allergic rhinitis (AR), also known as rhinallergosis, is a type I allergic disease of the nasal mucosa mediated by IgE after atopic individuals are exposed to allergens.^[1]^ A so-called atopic individual refers to anyone with genes related to the pathogenesis of AR, and they have a tendency to produce IgE antibody responses to common antigens in the environment and are susceptible to allergic diseases such as atopic dermatitis and asthma.^[2,3]^ IgE can mediate the degranulation of mast cells and basophils in atopic individuals to produce a large number of inflammatory mediators, leading to the occurrence of type I allergy. Consequently, AR patients develop discomforts namely repeated sneezing, a running nose with profuse watery mucus, nasal congestion, and nasal itching. Some patients may also be accompanied by eye itching, conjunctival hyperemia, and/or lacrimation.^[4,5]^ AR affects 10%–40% of the world’s population, and about 600 million people worldwide are victims of AR, and its prevalence keeps increasing without any effective cure to curb the development of this trend.^[6]^ Although AR is not life-threatening, the nasal and/or ocular symptoms due to AR will seriously affect the quality of life of patients and bring serious psychological pressure on them, which has become a huge burden on individuals and society as well as a global health problem.^[7,8]^

The treatment principles of AR include avoidance of allergens, pharmacotherapy, allergen immunotherapy, and education of patients.^[9]^ Although the avoidance of allergens is the most direct, effective, and economical treatment for AR, most allergens are inhaled allergens, such as dust mites, pollen, and animal dander, which are often difficult to achieve “zero” contact in real life.^[10]^ The typical treatment methods for AR are still medication therapy and specific immunotherapy (SIT).^[11]^ The medication therapy takes into effect by blocking the related inflammatory mediators such as histamine and leukotrienes produced during the pathogenesis of AR, including mast cell stabilizers that can stabilize mast cell membranes and prevent mast cell degranulation, H1 receptor antagonist against histamine effect and leukotriene receptor antagonist against leukotriene effect. Although the medication therapy has a rapid onset of action and can effectively improve the nasal and ocular symptoms of AR patients,^[12,13]^ it does not have long-term efficacy as a symptomatic treatment, and the patient’s symptoms will recur after the medication discontinues. Unfortunately, long-term medication may also cause patients to develop drug resistance or develop side effects.^[14]^ Therefore, as the only treatment method for AR at present, the role and status of SIT in AR management are gradually gaining more attention.

Intralymphatic immunotherapy (ILIT) is modified from the SIT method. It injects an allergen vaccine into the peripheral superficial lymph nodes of AR patients under the precise positioning of B-ultrasound, to improve body response to the allergen vaccine in AR patients. The efficiency of the immune response can rapidly induce the immune tolerance of the corresponding allergen in the body, so as to achieve the efficacy of immunotherapy.^[15,16]^ This method requires patients to receive only 3 shots of lymph node injections, with an interval of 28 days between each shot, and it takes only 2 months to complete a total desensitization treatment. However, there is still controversy about ILIT against AR. A meta-analysis^[17]^ has found that ILIT is not an effective way to control AR. It is therefore that the current research aimed to resolve the controversy by summarizing the latest randomized control studies and providing new options for patients with AR.

## 2. Methods and data

The study was registered with PROSPERO (CRD42022375992) and followed PRISMA-P (the preferred reporting project for system review and meta-analysis scheme) guidelines.^[18]^

### 2.1. Literature retrieval

The electronic databases PubMed, Embase, Cochrane library, and Web of Science were employed to retrieve randomized control studies on ILIT against AR, and the search deadline was September 15, 2023. The key words for search strategies included Injections, Intralymphatic, Endolymphatic Injection, Intralymphatic immunotherapy, Rhinitis, Allergic and Allergic Rhinitides. The detailed PubMed retrieval strategies are listed in Table S1, Supplemental Digital Content, http://links.lww.com/MD/N989.

### 2.2. Inclusion criteria

The patients who met the diagnostic criteria of AR,^[19]^ aged > 18, and accepted ILIT, were enrolled in the experimental group,^[20]^ while those who took a placebo or conventional western medicine for AR were included in the control. The main outcome indicators were symptom score (SS), medication score (MS), comprehensive symptom and medication score (CSMS), and adverse events. The secondary indicators were rhinoconjunctivitis Quality of Life Questionnaire. Randomized control studies that fulfilled the above-described criterion were considered for inclusion.

### 2.3. Data extraction

Two authors independently extracted the basic characteristics of the included studies, and if no outcome data were available, the corresponding authors were contacted. The extracted information included: first author, year of publication, type of study, sample size, allergen, age, intervention, injection site, follow-up time, and outcome indicators.

### 2.4. Quality appraisal

Two investigators independently assessed the risk-of-bias using Version 2 of the Cochrane risk-of-bias tool for randomized trials (RoB2),^[21]^ including the following 5 aspects: randomization process, deviation from intended interventions, missing outcome data, outcome measures, and choice of reporting results. For each major study, the risk-of-bias was rated as low, high, or unclear. If there is a dispute in some of the judgments, then the dispute will be discussed.

### 2.5. Statistical analysis

Standardized mean difference (SMD) was used for continuous data results, risk ratio (RR) was used for dichotomous data results, and precision values were reported with 95% confidence intervals for each variable. Due to large clinical heterogeneity among the included studies, a random effects model was applied for pooled data analysis, and Cochran *Q* and *I*^2^ statistics were employed to assess statistical heterogeneity, the *I*^2^ values of 25% to 50%, 51% to 75%, and 76% to 100% were preliminarily classified as low, medium and high, respectively. If heterogeneity was >50%, sensitivity analysis was applied to explore the specific source of heterogeneity. In addition to selection bias, the determination of publication bias was also detected using Egger test. *P* > .05 indicated that there was no publication bias detected. We calculated the change in the score using the method recommended in the Cochrane Handbook (the correlation between baseline and endpoint measures was 0.5).^[22]^ Data analysis was performed using Stata 15.0 software.

## 3. Results

### 3.1. Literature screening and results

**Figure 1.**
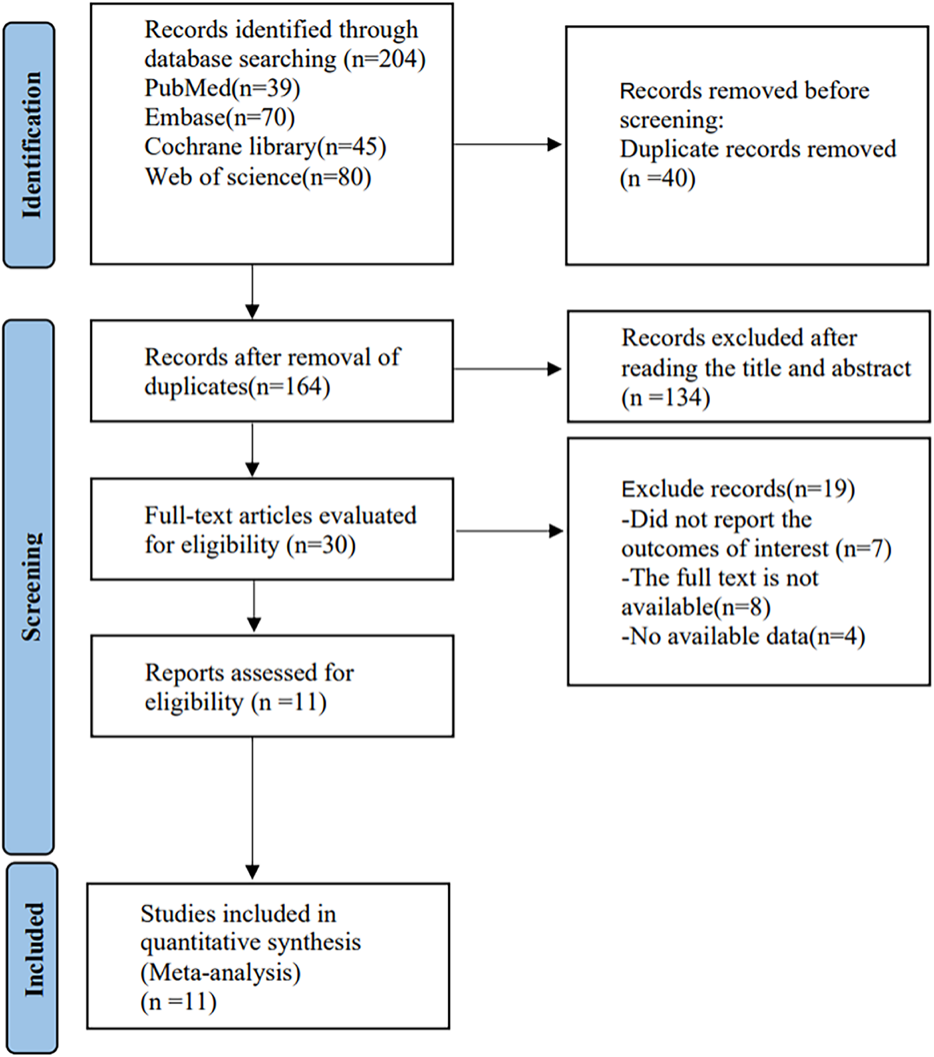
Literature search flowchart.

Figure 1 presents the retrieved 204 studies from the electronic database by data search. There were 164 articles were left by removing duplicate literature. After preliminary screening by reading titles and abstracts, and double screening by reading through full texts, a total of 11^[23–33]^ randomized control trials were finally included.

### 3.2. Baseline table of the included literature

A total of 11 randomized control studies were included involving 406 cases, most of which were from Sweden. The control group by Senti 2008^[23]^ applied subcutaneous immunotherapy (SCIT), while a majority of studies adopted inguinal immunotherapy. The baseline table of the included studies is shown in table 1.

**Table 1:** Basic features of literature.

Study	Country	Study design	Sample size (Male)	Allergen	Age (year)		Intervention		ILIT injection site	Follow-up (W)	Outcome
					EG	CG	EG	CG			
Hellkvist 2022	Sweden	RCTs	35 (22)	grass pollen	NA	NA	ILIT	Placebo	NA	NA	F1; F2; F3; F4; F6; F11
Hjalmarsson 2022	Sweden	RCTs	34 (23)	Birch; grass	35	31	ILIT	Placebo	NA	5 year	F1; F2; F3; F6; F11
Senti 2012	Sweden	RCTs	20 (6)	cat dander	34.6	27	ILIT	Placebo	NA	48	F4;
Hellkvist 2018	Sweden	RCTs	51 (35)	Birch; grass	35 ± 8.4	31.5 ± 6.9	ILIT	Placebo	Inguinal	24–36	F2; F3; F5; F9; F10
Hylander 2013	Sweden	RCTs	15 (10)	Birch; grass	35.25 ± 7.25	32.5 ± 10.02	ILIT	Placebo	Inguinal	NA	F3; F4; F5
Hylander 2016	Sweden	RCTs	36 (22)	Birch; grass	33 ± 8	33 ± 9.39	ILIT	Placebo	Inguinal	4	F4; F5;
Park 2021	Korea	RCTs	32 (13)	Hair dogs and cats.	32.4 ± 11.1	36.9 ± 5.9	ILIT	Placebo	Inguinal	48	F1; F2; F5; F10
Konradsen 2020	Sweden	RCTs	26 (16)	birch; timothy	24.25 ± 6.5	22 ± 5	ILIT	Placebo	Inguinal	NA	F1; F2
Senti 2008	Sweden	RCTs	112 (73)	grass pollen	32 ± 8.7	36 ± 12	ILIT	SCIT	Inguinal	NA	F5; F10
Skaarup 2021	Denmark	RCTs	24 (9)	grass pollen	29.92 ± 6.8	30.58 ± 7.43	ILIT	Placebo	Inguinal	144	F4; F12
Thompson 2020	American	RCTs	21 (9)	mountain cedar pollinosis	35 ± 12	40 ± 13	ILIT	Placebo	Inguinal	NA	F1; F3; F4; F6; F11

RCTs = randomized controlled trial, EG = experimental group, CG = control group, ILIT = intralymphatic immunotherapy, SCIT = subcutaneous immunotherapy, F1 = symptom score, F2 = quality of life (QOL)/RQLQ, F3 = serum index, F4 = adverse reactions, F5 = VAS scores, F6 = medication score, F7 = therapeutic effect, F8 = Th1/Th2 cell level, F9 = SPT skin prick test, F10 = used rescue medication, F11 = CSMS combined symptoms and medication score, F12 = provocation tests.

### 3.3. Quality appraisal

All included trials were carried out and strictly followed the study design, including the use of appropriate randomization methods and concealment, thus the aspect of randomization showed a low risk-of-bias. Most of the biases were due to blinding the outcome assessment researchers. The risk-of-bias assessment of the included studies is shown in Figures S1 and S2, Supplemental Digital Content, http://links.lww.com/MD/N989.

### 3.4. Meta-analysis results

#### 3.4.1. Symptom score

**Figure 2.**
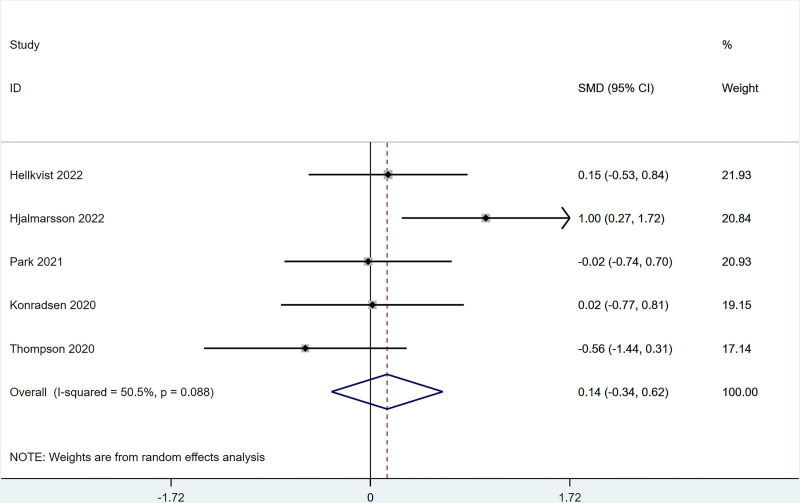
Meta-analysis forest map of symptom score (SS).

Five studies reported that SS change after ILIT treatment against AR did not differ greatly from the control group [SMD = 0.14, 95% CI = (‐0.34, 0.62)]. The heterogeneity test (*I*^2^ = 50.5%, *P* = .088) indicated the presence of heterogeneity among studies, see Figure 2. For the heterogeneity >50%, we performed a sensitivity analysis to figure out the source of the heterogeneity, and the results of the sensitivity analysis indicated that each study was within the interval, with low sensitivity and stable analysis results, see Figure S3, Supplemental Digital Content, http://links.lww.com/MD/N989.

#### 3.4.2. Quality of life

**Figure 3.**
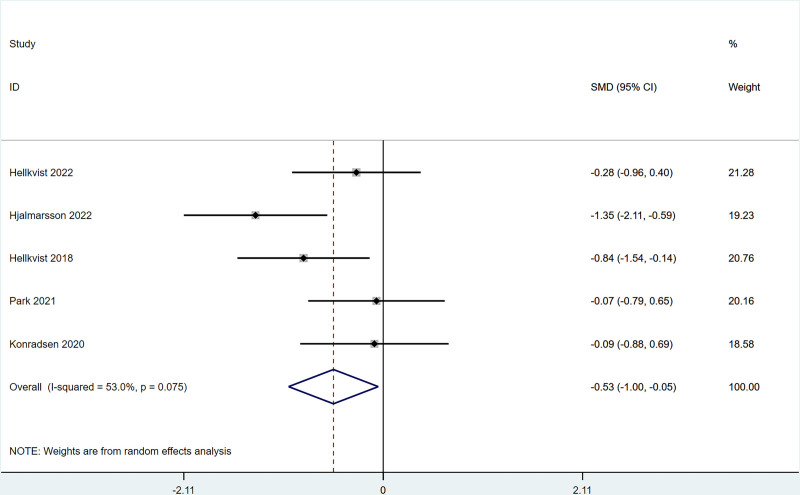
Meta-analysis forest map of quality of life (QOL).

There were 5 studies mentioned changes in quality of life after ILIT treatment for AR, compared with the control group, the quality of life in the ILIT group was markedly improved [SMD = ‐0.53, 95% CI = (‐1.00, −0.050)]and heterogeneity tests (*I*^2^* *= 53%, *P* = .075) indicated the presence of heterogeneity among studies, Figure 3. For the heterogeneity >50%, we performed a sensitivity analysis to figure out the source of the heterogeneity, and the results of the sensitivity analysis indicated that each study was within the interval, with low sensitivity and stable analysis results, see Figure S4, Supplemental Digital Content, http://links.lww.com/MD/N989.

#### 3.4.3. Immunoglobulin E

**Figure 4.**
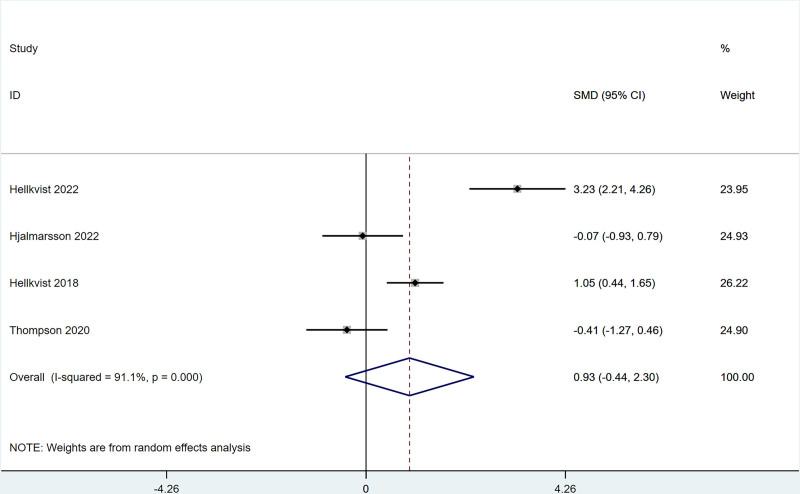
Meta-analysis forest map of IgE.

There were 4 studies that mentioned that IgE change after ILIT treatment against AR did not differ greatly from that of the control group [SMD = 0.93, 95% CI = (‐0.44, 2.30)]. The heterogeneity test (*I*^2^ = 50.5%, *P* = .001) indicated the presence of heterogeneity among studies, see Figure 4. For the heterogeneity >50%, we performed a sensitivity analysis to figure out the source of the heterogeneity, and the results of the sensitivity analysis indicated that each study was within the interval, with low sensitivity and stable analysis results, see Figure S5, Supplemental Digital Content, http://links.lww.com/MD/N989.

#### 3.4.4. Medication scores

**Figure 5.**
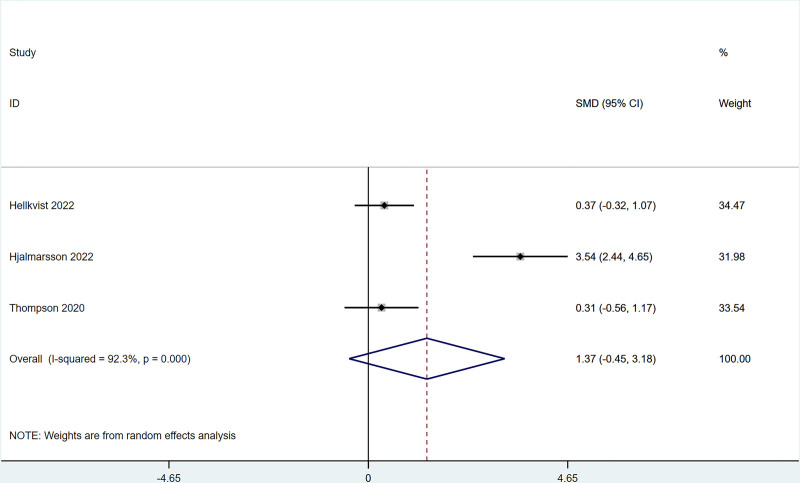
Meta-analysis forest map of medication scores (MS).

There were 3 studies that reported MS changes after ILIT treatment against AR that did not differ greatly from that of the control group [SMD = 1.37, 95% CI = (-0.45, 3.18)]. The heterogeneity test (*I*^2^ = 92.3%, *P* = .001) indicated the presence of heterogeneity among studies, see Figure 5.

#### 3.4.5. Comprehensive symptom and medication score

**Figure 6.**
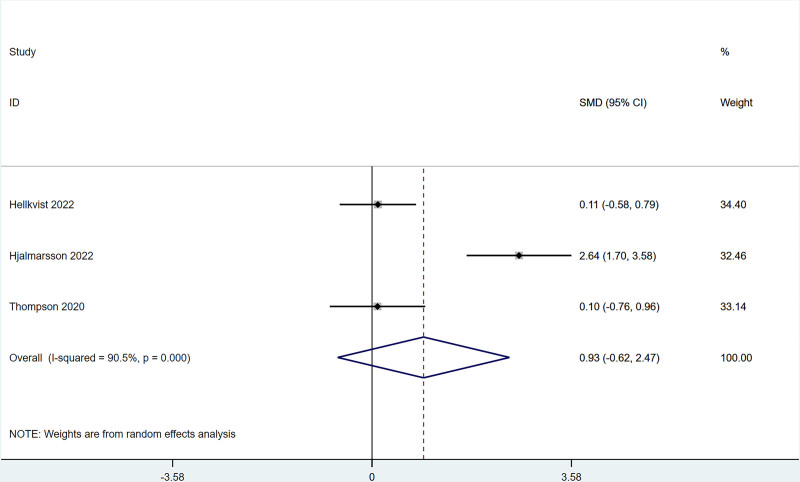
Meta-analysis forest map of composite symptom and medication scores (CSMS).

There were 3 studies that reported CSMS changes after ILIT treatment against AR that did not differ greatly from that of the control group [SMD = 0.93, 95% CI = (‐0.62, 2.47)]. The heterogeneity test (*I*^2^ = 90.5%, *P* = .001) indicated the presence of heterogeneity among studies, see Figure 6.

#### 3.4.6. Adverse events

**Figure 7.**
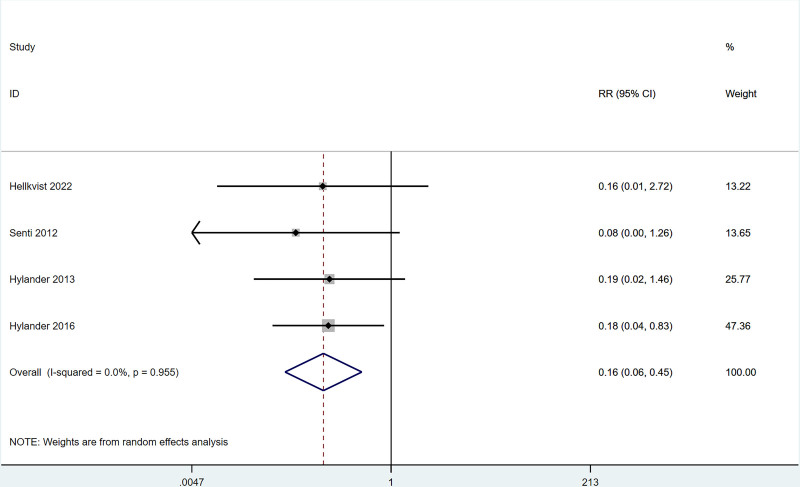
Meta-analysis forest map of adverse events.

**Figure 8.**
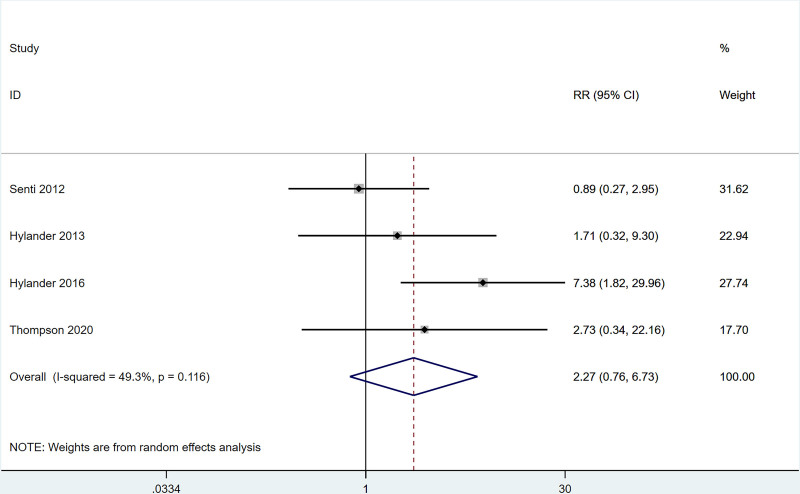
Meta-analysis forest map of incidence of lymphadenopathy.

Four studies reported that the incidence of nasal symptoms (including nasal congestion, itching, runny nose) after ILIT treatment of AR was lower than that in the control group [RR = 0.16, 95% CI = (0.06, 0.45)]. The heterogeneity test (*I*^2^ = 0%, *P* = .995) indicated the presence of heterogeneity among studies, see Figure 7. Four studies reported that the incidence of lymphadenopathy after ILIT treatment of AR was not markedly different from that of the control group [RR = 2.27, 95% CI = (0.37, 6.73)]. The heterogeneity test (*I*^2^ = 49.3%, *P* = .116) indicated the absence of heterogeneity among studies, see Figure 8.

### 3.5. Publication bias

The Egger test was applied to evaluate the publication bias of SS and quality of life, with values of *P *= .305 and *P* = .885 respectively, implying the publication bias of both SS and quality of life was not detected, as shown in Figures S6 and S7, Supplemental Digital Content, http://links.lww.com/MD/N989.

## 4. Discussion

Although previous studies^[17,34]^ have been published on similar topics, this meta-analysis added new findings or knowledge that resulted in additional changes or information. Intralymphatic immunotherapy (ILIT) is practically a modified specific immunotherapy method, which allows the injection of an allergen vaccine into the peripheral superficial lymph nodes of AR patients under the precise positioning of B-ultrasound, to improve body response to allergen vaccine in AR patients. The efficiency of the immune response can rapidly induce the immune tolerance of the corresponding allergen in the body, to achieve the efficacy of immunotherapy.^[35]^ Johansen et al^[36]^ confirmed through animal experiments that direct injection of an allergen vaccine into the superficial lymph nodes of rabbits can greatly improve the efficiency of the allergen adaptive immune response. Compared with subcutaneous injection (SCIT), the immune effect of ILIT can be enhanced by up to 10^6^ times.

The present work revealed that after ILIT treatment, the quality of life of AR patients was markedly improved [SMD = ‐0.53, 95% CI = (‐1.00, ‐0.050)], and adverse events of nasal symptoms were reduced [RR = 0.16, 95% CI = (0.06, 0.45)], whereas there were no significant difference in SS [SMD = 0.14, 95% CI = (‐0.34, 0.62)], IgE [SMD = 0.93, 95% CI = (‐0.44, 2.30)], MS [SMD = 1.37, 95% CI = (‐0.45, 3.18)], CSMS [SMD = 0.93, 95% CI = (‐0.62, 2.47)], nasal symptoms [RR = 0.16, 95% CI = (0.06, 0.45)], and lymphadenopathy [RR = 2.27, 95% CI = (0.37, 6.73)] versus control. This is consistent with the conclusion of Lee et al^[37]^ in 2017. After the 11 AR cases underwent ILIT, although the dosage of glucocorticoids application was reduced, adverse reactions of the patients’ nasal symptoms were reduced, and the quality of life was improved, there were no significant changes in serum-specific IgG4 and IgE in patients’ allergic to animal dander. The molecular mechanism of ILIT is consistent with traditional specific immunotherapy, but the immune response induced by ILIT produces apparent advantages over traditional immunotherapy.^[38]^ First, lymph nodes are the principal habitat of mature T cells and B cells, and major sites of the immune response, which can only be induced when antigens enter into the lymph nodes and bind to specific antibodies.^[39]^ Only 3 shots of injections are needed in ILIT, and the course of treatment is shortened to 3 months, thereby reducing suffering from frequent injections and potential adverse reactions.^[40]^ AR causes patients to develop repeated sneezing, a large amount of watery mucus, nasal congestion, and nasal itching, and some patients may also be accompanied by eye itching, conjunctival hyperemia, and/or lacrimation. The described nasal and/or ocular symptoms seriously affect the quality of life, including daily life, sleep, work, and study of patients. Therefore, the evaluation of AR treatment efficiency should focus on the improvement of symptoms and changes in the quality of life of patients after treatment.^[41]^ Linder^[42]^ has demonstrated that SS, as a subjective symptom evaluation index, has high sensitivity and specificity in the assessment of rhinitis symptoms for the first time since 1998. Spector et al^[43]^ have pointed out that SS can objectively reflect the severity of symptoms and the quality of life of patients, exerting a vital role in the assessment of rhinitis conditions. This study employed factors of SS, MS, and CSMS, respectively, and found that there was no significant difference between the experimental group and the control group, which might be caused by the different follow-up times in each study.

This study still has the following limitations: First, the number of included studies was small with a limited number of included cases and most of them were from Europe. Second, the allergens of the included studies were not consistent, and the interventions applied in controls were also not consistent, which might affect our research conclusions. Third, the follow-up times of the included articles varied, plus the partial follow-up time was too short, it is therefore that the safety could not be fully evaluated.

## 5. Conclusion

After ILIT treatment, the quality of life of AR patients was improved and the incidence of adverse events of nasal symptoms was reduced, indicating that ILIT could be introduced as a promising treatment option for AR patients. Given the presence of high heterogeneity, more high-quality randomized control trials are needed to verify the conclusion of the current research in the future.

## Acknowledgments

We would like to thank the researchers and study participants for their contributions.

## Author contributions

**Conceptualization:** Liangrong Liu, Yacheng Liang, Zhiyong Li.

**Data curation:** Liangrong Liu, Yacheng Liang, Zhiyong Li.

**Formal analysis:** Liangrong Liu, Yacheng Liang, Le Yan, Zhiyong Li.

**Funding acquisition:** Liangrong Liu, Le Yan, Zhiyong Li.

**Investigation:** Liangrong Liu, Zhiyong Li.

**Project administration:** Liangrong Liu, Le Yan.

**Resources:** Liangrong Liu.

**Software:** Liangrong Liu, Yacheng Liang, Le Yan.

**Supervision:** Liangrong Liu, Yacheng Liang.

**Validation:** Liangrong Liu, Yacheng Liang, Le Yan.

**Visualization:** Liangrong Liu.

**Methodology:** Yacheng Liang.

**Writing – original draft:** Liangrong Liu, Yacheng Liang, Le Yan, Zhiyong Li.

**Writing – review & editing:** Zhiyong Li.

## Supplementary Material
