# Supplementary material for: Is intralymphatic immunotherapy effective and safe for allergic rhinitis?: A meta-analysis
Source: Medicine (Baltimore). 2024 Nov 15;103(46):e40589. doi: 10.1097/MD.0000000000040589 (PMC11576000; doi:10.1097/MD.0000000000040589)

SUPPLEMENTARY MATERIAL

Supplementary Tables

ID	
#1	"Rhinitis, Allergic"[Mesh]
#2	(((Rhinitis, Allergic[Title/Abstract]) OR (Allergic Rhinitides[Title/Abstract])) OR (Rhinitides, Allergic[Title/Abstract])) OR (Allergic Rhinitis[Title/Abstract])
#3	#1 OR #2
#4	"Injections, Intralymphatic"[Mesh]
#5	(((((((((Injections, Intralymphatic[Title/Abstract]) OR (Injections, Endolymphatic[Title/Abstract])) OR (Endolymphatic Injections[Title/Abstract])) OR (Endolymphatic Injection[Title/Abstract])) OR (Injection, Endolymphatic[Title/Abstract])) OR (Intralymphatic Injections[Title/Abstract])) OR (Injection, Intralymphatic[Title/Abstract])) OR (Intralymphatic Injection[Title/Abstract])) OR (ILIT[Title/Abstract])) OR (Intralymphatic immunotherapy[Title/Abstract])
#6	#5 OR #6
#7	#3 AND #6

Table S1 Search strategies in PubMed.

Table S1 Search strategies in PubMed.

Supplementary Figure

FigureS1 Risk bias of graph

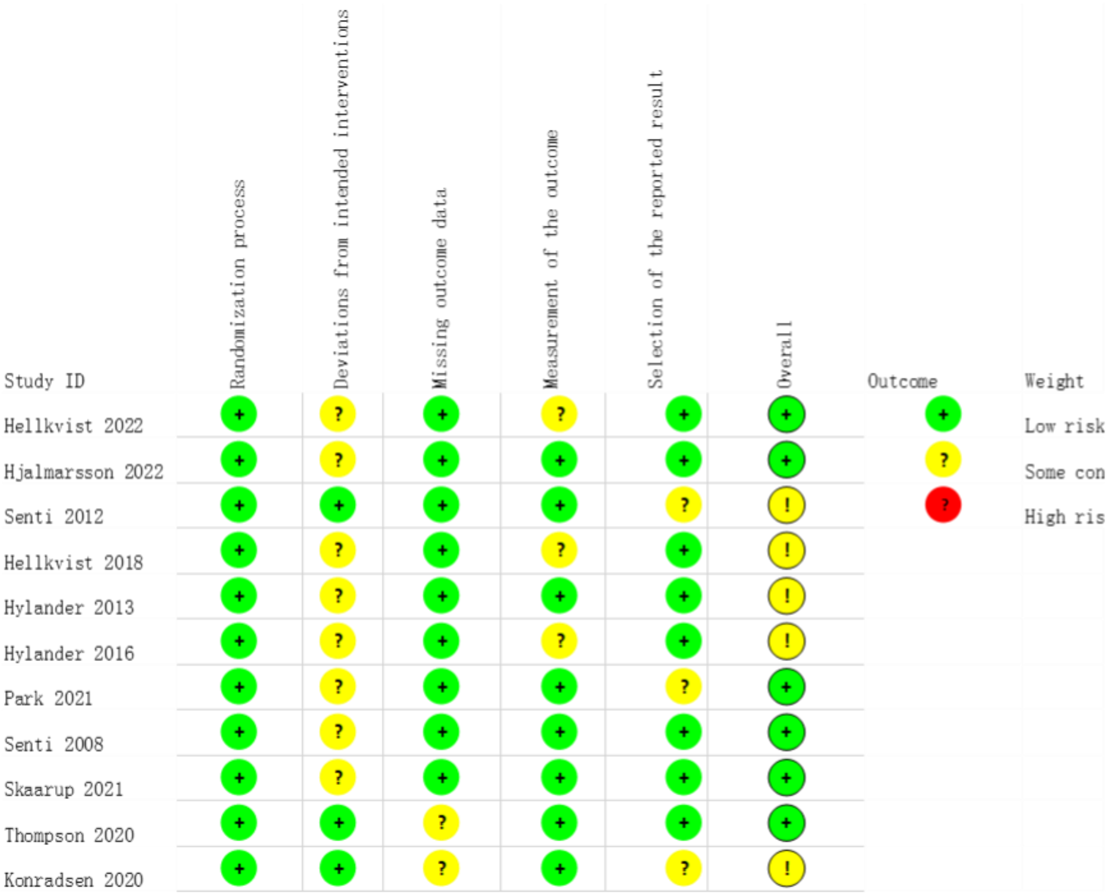

Figure S2 Risk bias of summary

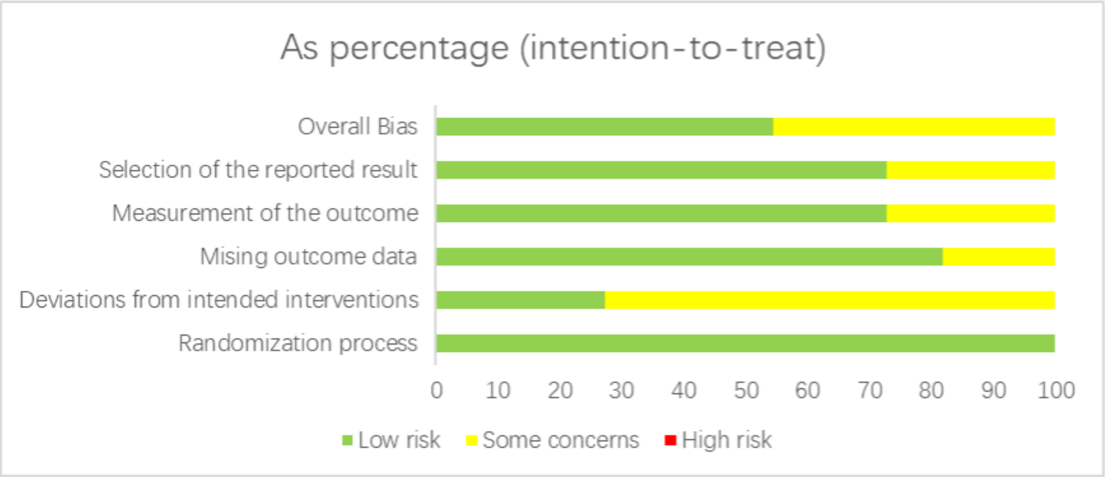

FigureS3 Sensitivity analysis of symptom score (SS)

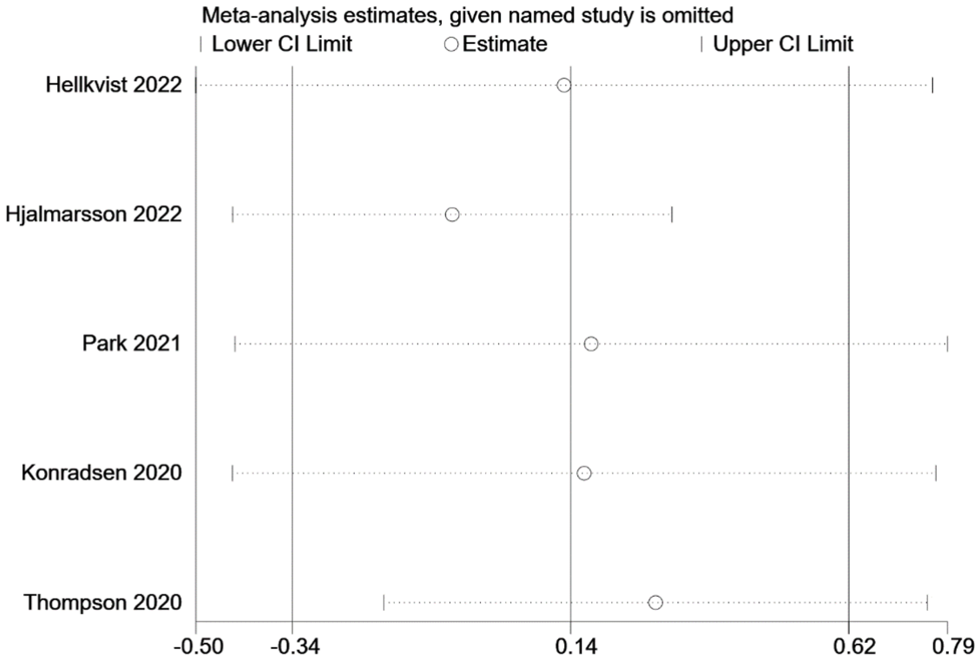

FigureS4 Sensitivity analysis of quality of life (QOL)

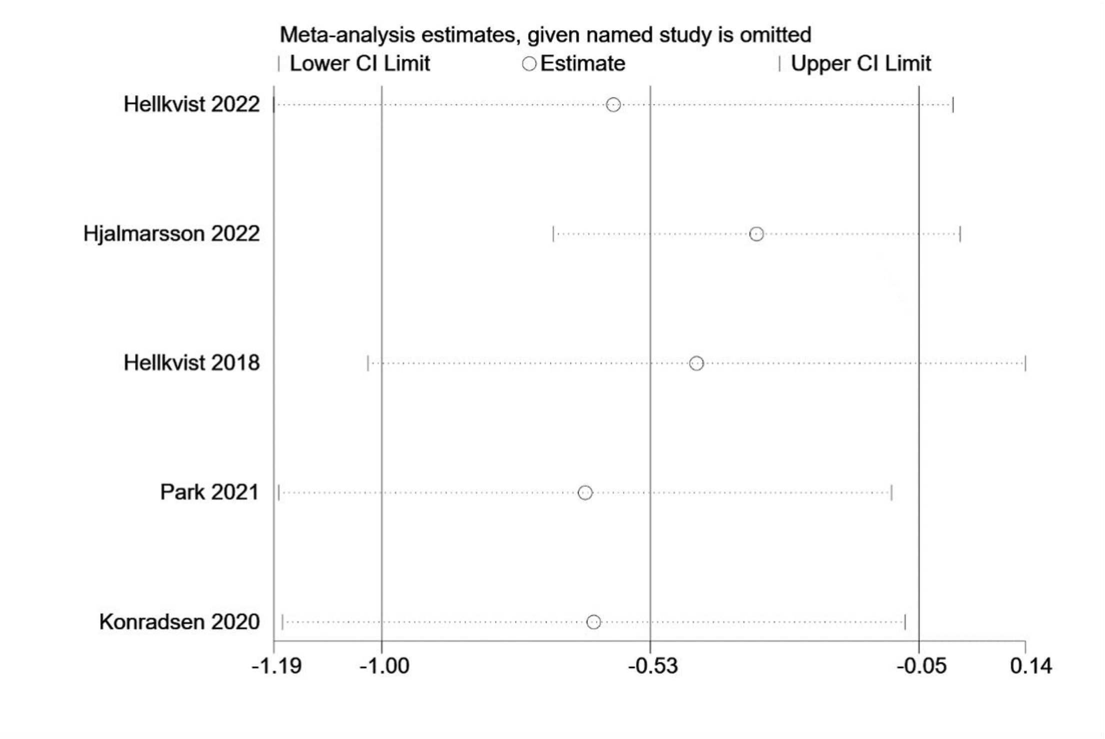

FigureS5 Sensitivity analysis of IgE

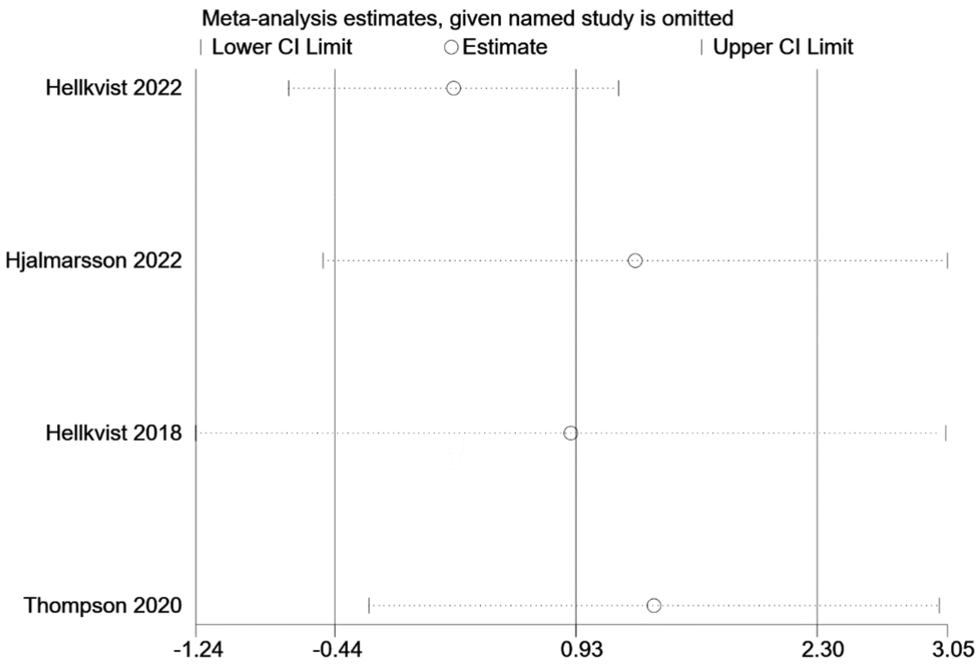

Figure S6 Egger test for symptom score (SS)

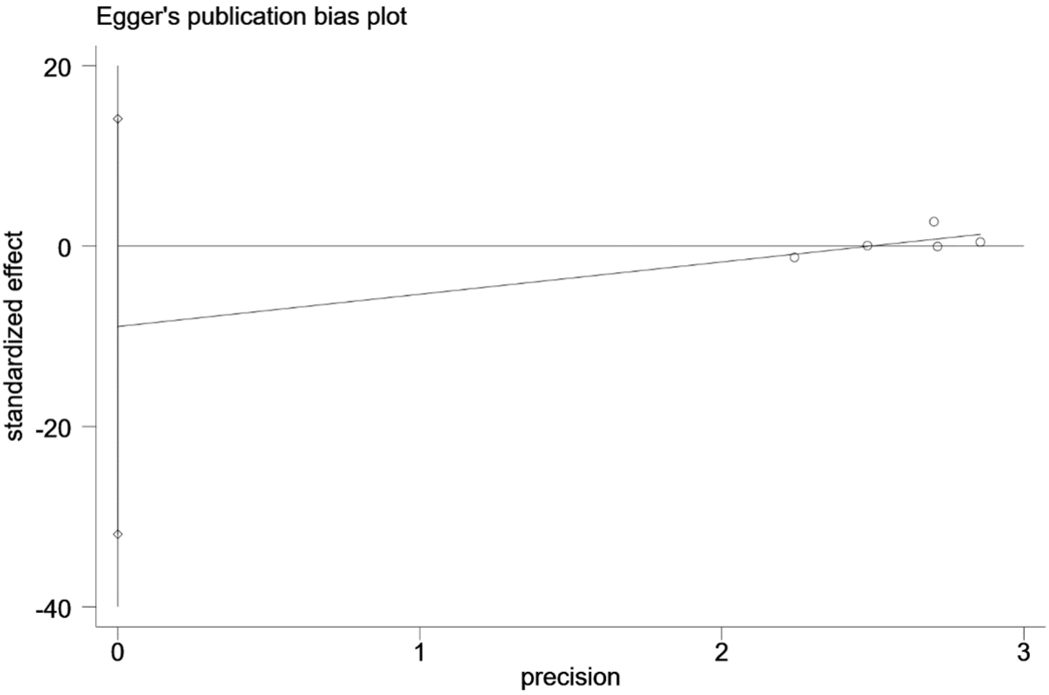

Figure S7 Egger test for quality of life (QOL)

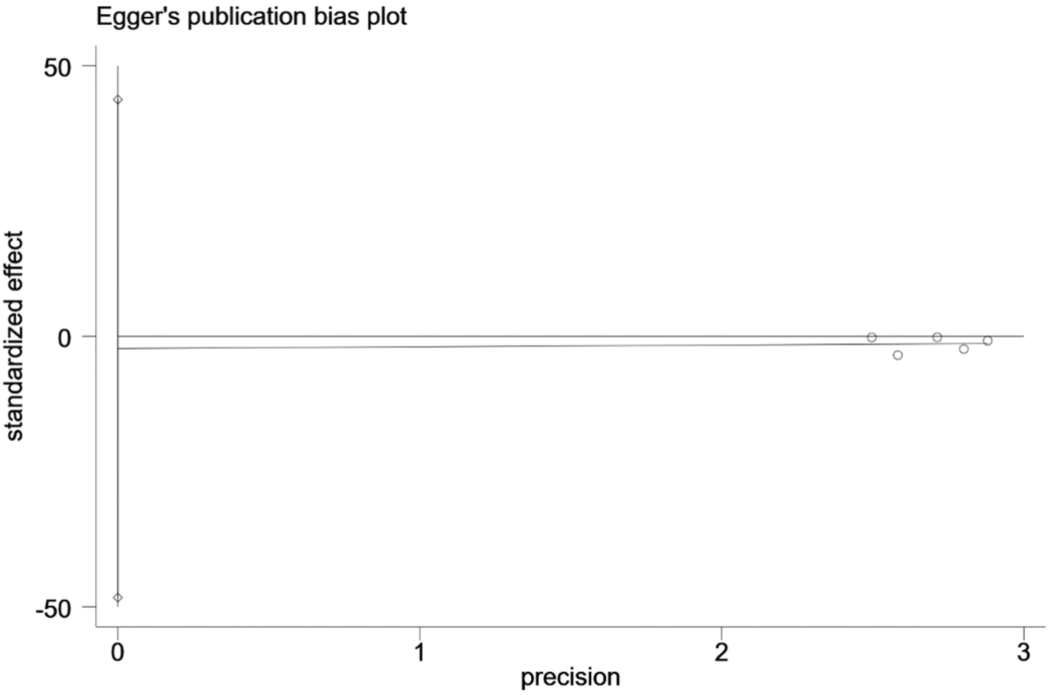

Supplement: Supplementary file 1 [file medi-103-e40589-s001.pdf]
